# Comparison of Morphological and Functional MRI Assessments of Periprostatic Fat for Predicting Prostate Cancer Aggressiveness

**DOI:** 10.1590/S1677-5538.IBJU.2024.0318

**Published:** 2025-01-10

**Authors:** David Freire Maia Vieira, Cecília Vidal de Souza Torres, André de Freitas Secaf, Matheus de Moraes Palma, Gabriel de Lion Gouvea, Elias Jorge, Rodolfo Borges Reis, Valdair Muglia

**Affiliations:** 1 Universidade de São Paulo Faculdade de Medicina de Ribeirão Preto Departamento de Imagem Ribeirão Preto SP Brasil Departamento de Imagem, Oncologia e Hematologia - Hospital das Clínicas da Faculdade de Medicina de Ribeirão Preto, Universidade de São Paulo - USP, Ribeirão Preto, SP, Brasil; 2 Universidade de São Paulo Faculdade de Medicina de Ribeirao Preto Departamento de Cirurgia e Anatomia Ribeirão Preto SP Brasil Departamento de Cirurgia e Anatomia, Faculdade de Medicina de Ribeirao Preto, Universidade de São Paulo, Ribeirão Preto, SP, Brasil; 3 Universidade de São Paulo Clínica Médica Faculdade de Medicina de Ribeirão Preto Ribeirão Preto SP Brasil Clínica Médica Faculdade de Medicina de Ribeirão Preto - Universidade de São Paulo - USP, Ribeirão Preto, SP, Brasil

**Keywords:** Morphological and Microscopic Findings, Magnetic Resonance Imaging, Diagnosis

## Abstract

**Purpose:**

The objective of this study was to evaluate whether morphological (linear measurements) and functional (ADC value) assessments of periprostatic fat can predict the aggressiveness of prostate cancer (PCa) over a 5-year follow-up period.

**Material and Methods:**

This retrospective study included patients with histologically proven PCa who underwent 3.0T MRI between July 2016 and June 2018. Clinical and demographic data collected included PSA, PSA density (dPSA), ISUP grade, clinical and pathological staging, and treatment details. MRI-derived parameters were assessed by an experienced radiologist, who measured subcutaneous and periprostatic fat thickness, and calculated ADC values from ROI plots in periprostatic fat. Clinical and MRI parameters were analyzed for associations with biochemical recurrence, systemic metastasis, and PCa-related mortality.

**Results:**

After applying exclusion criteria, 109 patients were included. Using the Cox model, dPSA (p<0.01), systemic disease at diagnosis (p<0.01), and mean ADC (p<0.02) were independent predictors of overall survival (OS). For progression-free survival (PFS), only dPSA (p<0.01) and systemic disease at diagnosis (p<0.01) were significant predictors. In the Poisson Model for systemic recurrence risk, dPSA had a relative risk (RR) of 1.04 (95%CI 1.0-1.07, p=0.03), systemic disease at diagnosis had an RR of 63.3 (95%CI 3.7-86.4, p<0.01), and average ADC had an RR of 3.42 (95%CI 1.52-7.69, p<0.01).

**Conclusions:**

The ADC value of periprostatic fat may serve as an additional tool for PCa risk stratification, correlating with poorer outcomes such as systemic recurrence and overall survival. If validated by external, prospective, multicenter studies, these findings could impact future therapeutic decisions.

## INTRODUCTION

Prostate cancer (PCa) is the most common non-cutaneous cancer in men and the fourth leading cause of cancer mortality in men worldwide. In 2020, over 1.4 million new cases of prostate cancer were reported globally (1).

A wide variety of external and environmental factors are associated with the risk of developing PCa (2, 3). Specifically, different eating habits and genetic susceptibilities have been shown to influence the risk of PCa (4). Family history, BRCA gene mutations, and metabolic syndrome (especially hypertension and obesity) are associated with a higher risk of developing PCa (5). Dietary factors, such as alcohol intake, have also been linked to a higher risk of PCa and PCa-related mortality (6). Therefore, preventive interventions at all levels of care may influence adherence to disease treatment and prevention of its progression.

Currently, the diagnosis of PCa is based on prostate biopsy, preferably after a Prostate MRI has identified a lesion (7-10). The fusion-guided US/MRI biopsy has become the modality of choice, significantly enhancing the detection of clinically significant PCa, while reducing the detection of clinically insignificant cases (11). In addition to the development of new software, artificial intelligence models have increased the positive predictive value of fusion biopsies compared to those using MRI alone (12). Also, over the past decade, liquid biopsy has been extensively studied as a non-invasive alternative for detecting and predicting prostate neoplasia, primarily through the quantification of serum biomarkers (13).

Multiparametric magnetic resonance imaging (mpMRI) is used beyond diagnosis to identify the extent of extra prostatic involvement, invasion of neurovascular structures, and for stratification of clinically significant lesions, with high accuracy (14).

The apparent diffusion coefficient (ADC) is an MRI quantitative parameter that calculates the extent of diffusion of water molecules within tissues using automatic software applied to MRI with diffusion-weighted imaging (DWI) (15). ADC measurement can be performed on a single slice by selecting a specific region of interest (ROI) in the relevant finding displayed on the map or can be volumetric when the entire lesion is evaluated (16). Low ADC values can indicate regions of high cellularity. Previous studies have shown that the ADC of periprostatic tissue is lower in patients with prostate cancer than in those without cancer and that there is an inverse correlation between ADC of the PCa and the Gleason score, which is an indicator of tumor aggressiveness (14).

The relationship between obesity and more aggressive cancers has been reported in many studies across various primary sites (17). The increase in serum growth factors and levels of pro-inflammatory cytokines resulting from obesity may explain this association (18). García-Martínez et al. also identified a relationship between the telomere ratio of subcutaneous and visceral fat as a possible biomarker in colorectal carcinoma (19). Furthermore, obesity has been linked to an increase in periprostatic adipose tissue, playing a specific role in the induction and progression of prostate neoplasia (20). The thickness of periprostatic and subcutaneous fat has been reported as a potential predictor of unfavorable outcomes in patients with prostate cancer, as it indirectly indicates a higher body mass index and greater risk of recurrence and mortality (21).

While the prognosis of prostate cancer can rely on several factors, the stage of the disease at diagnosis is a primary predictor (22). Most patients have low-risk, non-life-threatening tumors, but some have extremely aggressive disease, compromising their quality of life and leading to high rates of morbidity and mortality (23).

Current therapeutic options depend on the severity and extent of the disease and range from minimal or non-invasive methods, such as active surveillance (AS), to more aggressive treatments like radical prostatectomy, external radiotherapy/brachytherapy, chemical or surgical castration, and chemotherapy (24, 25). Identifying tumors with an unfavorable prognosis that present as localized disease at diagnosis could benefit patients through more aggressive therapeutic approaches (26).

Since functional changes usually precede morphological alterations, the objective of this study was to evaluate whether functional assessment (ADC value) of periprostatic fat is a better predictor of the aggressiveness of PCa than morphological assessment (linear measurements) over a 5-year follow-up.

## MATERIAL AND METHODS

### Patient Selection

This retrospective study was conducted at a single center with a waiver for informed consent due to its retrospective nature (IRB number 69251723.3.0000.5440). Using electronic data from the Radiology and Pathology records, we searched for "prostate cancer" and "prostate neoplasia" in both data sets, retrospectively identifying all patients with these diagnoses, confirmed by biopsy and/or surgery, who had undergone 3.0T multiparametric MRI between July 2016 and June 2018. This approach allows the study to evaluate each patient's outcomes over the 5-year oncological follow-up, including overall survival, disease-free period, biochemical recurrence, emergence of metastases, lymph node disease, and disease-related deaths. We also reviewed the electronic medical records of all patients, verifying their age, race, PSA levels, multiparametric MRI results, pathology (biopsy or surgical specimen), and treatments used.

**Figure 1:**
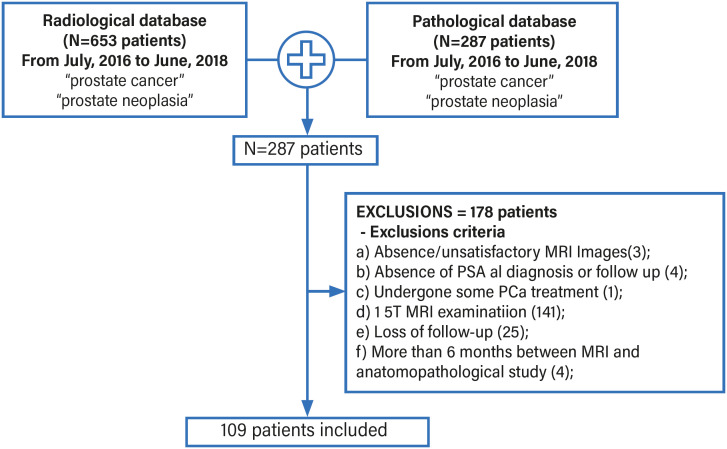
Flowchart showing database, the exclusions and final number of patients enrolled in this cohort.

The exclusion criteria were: a) absence/unsatisfactory MRI images; b) absence of PSA at diagnosis or follow-up; c) patients who had already undergone some PCa treatment; d) patients with 1.5T MRI examination; e) loss of follow-up; f) interval between MRI and anatomopathological study greater than 6 months (Figure-1).

### MRI Protocol

All MRI exams were performed using a 3 Tesla MRI machine, Achieva, manufactured by Philips (The Best- Netherlands), with a 16-channel Pelvic Phased Array coil. A protocol following PI-RADS v2.1 guidelines was employed (14), including high-resolution T2 acquisition in three planes, diffusion-weighted imaging (DWI) with b values of 0, 250, 500, and 1000 mm/s², and another acquisition with a b value of 1400 mm/s². Dynamic contrast enhancement (DCE) sequence was obtained with the injection of 0.1 mmol/kg gadolinium-based contrast agent, capturing images every 6 seconds for 10 minutes.

### MRI Analysis

**Figure 2:**
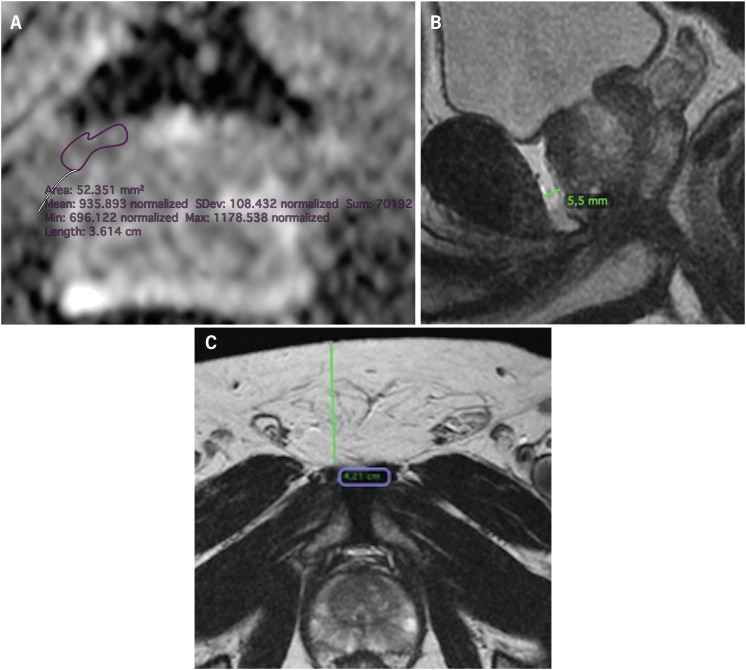
A) Measurement of the mean ADC value of periprostatic fat, using a ROI placed in the anterior periprostatic fat. B) Linear measurement of periprostatic fat thickness in the T2WI sagittal plane, and C) Linear measurement of subcutaneous fat thickness in the T2WI axial plane.

The PI-RADS classification was obtained from the original report and was based on a double reading by an experienced reader (15 years of prostate imaging) and one of four fellows in abdominal imaging. All images were reviewed by a radiologist with 5 years of experience for the quantitative analysis. The mean ADC value was calculated in the axial plane, with a circular ROI with an average diameter of 0.5 cm in the periprostatic fat between the prostatic base and the pubis (Figure-2A). Linear measurements of subcutaneous and periprostatic fat were performed in the axial and sagittal T2 plane using the same anatomical parameters as the ADC (Figures 2B and 2C).

The gold standard was histopathological analysis, either by biopsy (n=38 patients) or radical prostatectomy specimen (n=71 patients). Prostate biopsies were performed by attending physicians using cognitive or automated fusion, given the patients had prior MRI, using transrectal approach. For patients with both biopsy and prostatectomy, the analysis prioritized the surgical specimen for higher accuracy in locating index lesions. The correlation between tumor location in the surgical specimen/biopsy and MRI images was performed by the senior researcher with around 20 years of abdominal imaging experience.

Electronic medical records were analyzed by four radiologists with 4 years of experience, collecting the following information: race, age at diagnosis, PSA and dPSA at diagnosis, treatment performed, clinical and surgical staging, biochemical recurrence, disease progression within 60 months (metastasis or lymph node disease), and survival in 60 months.

### Statistical Analysis

Initially, the data were described using absolute frequencies and percentages (qualitative variables) and through measures such as mean, standard deviation, minimum, median, and maximum (quantitative variables). To investigate the potential association between periprostatic and subcutaneous fat and worse prognosis (death or recurrence), the Cox proportional hazards model was proposed (27). This model calculates the Hazard Ratio (HR), which indicates the risk of death/relapse in one category compared to another. To estimate the crude and adjusted Relative Risk, the Poisson regression model with robust variance (28), simple and multiple, was used.

All data analysis was performed using R software, version 4.1.3, or SAS 9.4. A significance level of 5% was adopted for all analyses.

## RESULTS

Initially, 287 patients were identified as having undergone mpMRI of the prostate and had a histopathological diagnosis of prostate cancer from July 2016 to June 2018. After applying the exclusion criteria, only 109 patients remained.

The mean age of patients was 65.3 ± 8.4 years. PSA levels ranged from 0.7 to 1346 ng/dL, with a median of 8.0. The PSA density (dPSA) varied from 0.04 to 72.8, with a median of 0.25 and a mean of 1.87 ± 8.8 (Table-1).

**Table 1:** Demographic, clinical and pathological data and MRI-derived parameters of the entire cohort.

PARAMETER			
Age (years			65.3 ± 8.4 (39-86)
PSA (ng/dL)			median - 8.04 (0.7-1346)
PSA density			median 0.25 (0.04-44.9)
		1	33 (30.3%)
		2	34 (31.2%)
ISUP		3	25 (22.9%)
		4	7 (6.4%)
		5	10 (9.2%)
		Negative	98 (89.9%)
Nodal Staging		Positive	11 (10.1%)
		Negative	101 (92.7%)
Systemic Metastases		Positive	8 (7.3%)
	I		8 (7.3%)
		A	21 (19.3%)
	II	B	39 (35.8%)
		C	2 (1.8%)
Clinical Staging		A	5 (4.6%)
	III	B	18 (16.5%)
		C	3 (2.7%)
		A	5 (4.6%)
	IV	B	8 (7.3%)
		Very Low	9 (8.2%)
		Low	5 (4.6%)
Risk Stratification		Favorable Intermediate	32 (29.4%)
		Non-Favorable Intermediate	23 (21.1%)
		High	40 (36.7%)
		Only Prostatectomy	58 (53.2%0
		Prostatectomy and combination*	16 (14.7%)
Therapeutics		Radiation Therapy and combination*	22 (20.2%)
		ADT - Androgen deprivation therapy	5 (4.6%)
		Active Surveillance	7 (6.4%)
		Orchiectomy and combination*	1 (0.9%)
**PARAMETER**			
Lesion size (mm)		15.7 ± 8.1 (5-42)	
Prostate Volume (cc)		median - 32.8 (12-307)	
Periprostatic fat (mm)		5.21 ± 2.96 (1.0 - 17.1)	
Subcutaneous fat (mm)		33.8 ± 13.5 (8-88)	
mean ADC (x10-3 mm/s2)		0.913 ± 0.319 (0.352-1.659)	
	2	16 (14.7%)	
	3	2 (1.8%)	
PI-RADS	4	44 (40.4%)	
	5	47 (43.1%)	

* and combination means association with chemotherapy and/or ADT.

Of the 109 PCa cases, 33 (30.3%) were non-significant cancers (GG6 / ISUP1), and 76 (69.7%) were clinically significant cancers with the following distribution: 34 (31.2%) had ISUP 2; 25 (22.9%) had ISUP3, and 17 (15.6%) had ISUP 4 or 5 PCa.

At the time of diagnosis, 78 (71.6%) had localized disease (T1 and T2), and 31 (28.4%) had extra prostatic disease, with 26 patients classified as T3 and 5 as T4. Regarding lymph node status, 98 were negative (89.9%) and 11 (10.1%) were involved. Eight patients (7.4%) had systemic disease at diagnosis (Table-1).

The MRI analysis indicated a high predominance of PI-RADS 4 (n=44, 40.4%) and 5 (n=47, 43.1%). Only 2 (1.8%) patients were scored as PI-RADS 3, and 18 (16.5%) had a score of 2. The quantitative analysis showed a mean thickness of periprostatic fat of 5.21 ± 2.96 mm (range 1.0 - 17.1 mm), while for subcutaneous fat, the values were 33.8 ± 13.5 mm (range 8 to 88 mm). The mean ADC of periprostatic fat was 0.913 ± 0.319 × 10^-3 mm/s². The mean size of cancerous visible lesions on mpMRI was 15.7 ± 8.1 mm, measured on the most conspicuous sequence (T2 or DWI) (Table-1).

When assessing patients’ outcomes, the overall survival was independently predicted by ISUP 2 or greater (at the limit, p=0.05), nodal (p<0.01), or systemic (p<0.01) involvement at the time of diagnosis. Notably, there was only one death among the 33 patients with ISUP 1 lesions (3.0%), while there were 14 deaths among the 76 patients (18.4%) with clinically significant cancers (ISUP 2 or greater).

For the assessment of the risk of recurrence using the Poisson Model, the independent predictors were nodal (p<0.01) or systemic (p<0.01) involvement and the mean ADC value of the periprostatic fat (p<0.01). In contrast, the thickness of periprostatic (p=0.29) or subcutaneous fat (p=0.11) were not predictors of recurrence (Table-2).

**Table 2:** Risk of systemic recurrence - Poisson Model and Overall survival - Cox model.

Risk of Systemic Recurrence: Poisson Model				
Characteristic	Relative Risk	CI 95%		p-value
PSA density (ng/ml/cm³)	01.04	1.00	01.07	**0.03**
PI-RADS: 4 and 5 vs 2 and 3	0.99	0.12	7.97	0.99
Staging: III/IV vs I/II	8.97	01.09	74.10	**0.04**
T3/T4 vs T1/T2	12.58	1.53	103.39	**0.02**
N1 vs N0	17.82	3.67	86.40	**<0.01**
M1 vs M0	63.13	8.35	477.17	**<0.01**
Periprostatic fat thickness (mm)	01.08	0.94	1.24	0.29
Subcutaneous fat thickness (mm)	0.92	0.84	01.02	0.11
Periprostatic adipose tissue ADC X 10-6	3.42	1.52	7.69	**<0.01**
Overall Survival Rates: Cox Model				
Characteristic	Hazard Ratio	CI 95%		p-value
ISUP grade group: 1 or >1	06.07	0.80	46.13	0.08
PSA density (ng/ml/cm³)	01.08	01.05	1.12	**<0.01**
PI-RADS: 4 and 5 vs 2 and 3	1.54	0.35	6.81	0.57
Staging: III/IV vs I/II	3.21	1.14	09.01	**0.03**
T3/T4 vs T1/T2	2.58	0.94	7.14	0.07
N1 vs N0	5.27	1.65	16.83	**0.01**
M1 vs M0	31.20	8.96	108.70	**<0.01**
Periprostatic fat thickness (mm)	01.05	0.88	1.25	0.60
Subcutaneous fat thickness (mm)	0.96	0.92	01.01	0.09
Periprostatic fat ADC X 10-6	2.74	1.15	6.51	0.02

p-values in bold are indicative of statistical significance (<0.05). Abbreviations: OR = Odds Ratio. CI = Confidence Interval; PSA. prostate-specific antigen; PIRADS. prostate imaging reporting & data system; ISUP. International Society of Urological Pathology.

The main outcome, overall survival, analyzed using the Cox model, showed statistical relevance for the ADC value of periprostatic fat (p<0.02) with a Hazard Ratio of 2.74 (1.15-6.51). There was no statistically significant value for periprostatic (p=0.60) and subcutaneous fat (p=0.09). These findings may indicate that patients who present functional and inflammatory changes in periprostatic fat may have a worse outcome than those without such changes (Table-2).

## DISCUSSION

Our results showed that dPSA, systemic disease at diagnosis, and mean ADC were independent predictors of overall survival (OS). For progression-free survival (PFS), only dPSA and systemic disease at diagnosis were significant predictors. These results are aligned with a recent study by Tafuri et al. (15), which reported higher positivity in biopsies and a higher ISUP grade in prostate neoplasms for patients with lower ADC values of periprostatic fat, indicating more aggressive tumors. Similarly, Gulcap et al. found that fat inflammation in periprostatic white adipose tissue, collected from 169 men undergoing prostatectomy, was associated with higher ISUP groups IV/V tumors (29). Despite some methodological and technical differences (1.5 T vs. our 3.0T scanner), we evaluated a similar number of patients (109 vs. 132), monitored clinical outcomes for a longer period (60 months vs. 30 months), and obtained comparable results.

Periprostatic fat has also been used in different contexts for patients with prostate cancer. Zhai et al. indicated that periprostatic fat measurement, combined with PI-RADS v2 scores, could predict pathologic upgrading among patients who had biopsy Gleason 6 (3+^3^) PCa after radical prostatectomy (30). A similar finding was reported by Uzun et al., who included all patients, not just those with non-clinically significant cancer (31). Gregg et al. suggested another interesting approach by assessing the periprostatic fat volume normalized by the prostate volume in patients under Active Surveillance. In their study, this parameter (normalized periprostatic fat volume) predicted which patients would have shorter progression-free survival (32).

Several mechanisms have been proposed for the association between obesity and prostate cancer (PCa). The role of periprostatic white adipose tissue as an important source of local factors that stimulate PCa progression, and it is a hot topic in research (3, 18). The adipocytes, and their progenitor stromal adipose cells (ASCs), which proliferate to accommodate the expansion of white adipose tissue in obesity, are being implicated as inductive factors of cancer progression. In preclinical studies, ASCs promote tumor growth by remodeling the extracellular matrix and supporting neovascularization (2).

However, the association between periprostatic fat and PCa aggressiveness has also been questioned in the literature. For instance, Laine-Caroff et al. did not find such an association in a cohort of 121 patients (33). In our study, linear measurement was not associated with aggressive tumors, similar as in the Laine-Caroff cohort. A potential interpretation is that morphological changes typically occur later in the disease course, and depending on the timing of MRI morphological measurements, they may not be fully developed, failing to demonstrate an association. Conversely, functional changes are usually more dynamic and occur earlier in any disease condition, allowing a functional parameter (like ADC) to demonstrate such associations almost from the early development of PCa and related periprostatic adipose tissue changes.

All these studies used morphological parameters, including periprostatic fat linear measurement, volume of periprostatic fat, subcutaneous fat, and/or variations such as normalized periprostatic fat volume. Despite being preliminary data and the need for further studies and validations, these promising results could form the basis for new studies evaluating the functional alteration of fat in patients with intermediate tumors, such as Gleason 3+4, to determine if they could benefit from more aggressive treatments.

There are some limitations to this study. It is a retrospective study from a single center with inherent problems, especially selection bias. We used strict inclusion and exclusion criteria to minimize these methodological limitations. We also had a limited number of patients and did not assess the reproducibility of results using a second reader, which also limits the generalizability of our results. However, our approach, using linear measurement of periprostatic fat and ROI placement for obtaining ADC values, are techniques extensively tested in the literature with consistent reproducibility (34, 35). Additionally, only one ADC value measurement was obtained, not a volumetric assessment. Contrary to tumors where a volumetric approach is more adequate for capturing heterogeneity and potential areas of undifferentiated tumors, adipose tissue without any medical intervention tends to be fairly homogeneous, making the ADC values difference between different levels of measurement negligible (16, 36). Lastly, our institution is a quaternary healthcare center with a high volume of oncological cases, which generates a selection bias in the studied population by pre-selecting more aggressive lesions.

In conclusion, our results indicate that the ADC value of periprostatic fat can be an additional tool for risk stratification in prostate cancer, as it was associated with worse outcomes (systemic recurrence and overall survival). If validated by external, prospective, and multi-institutional studies, these results could influence decisions regarding the therapeutic approach to these patients in the future.
